# Competition for nutrients or cell intrinsic programming? – Metabolic mechanisms behind the tumor promoting immune microenvironment in cancer

**DOI:** 10.1038/s41392-021-00693-2

**Published:** 2021-07-20

**Authors:** Yuequn Niu, Thomas Mayr, Michael H. Muders

**Affiliations:** Rudolf Becker Laboratory for Prostate Cancer Research, Center of Pathology, University Hospital Bonn, Bonn, Germany

**Keywords:** Cancer metabolism, Experimental models of disease

The prevention of anti-tumor immunity is a hallmark of cancer. Immune and cancer cells depend on glucose for their anabolic metabolism. Metabolic reprogramming of immune cells is essential to promote the growth of cancer cells. In a recent study published in *Nature*, Reinfeld et al.^1^ pinpointed the mTORC1 signaling pathway to be responsible for metabolic reprogramming of the immune cells in the tumor microenvironment (TME).

Unlike most normally differentiated cells, cancer cells are considered to be more dependent on glycolysis rather than mitochondrial oxidative phosphorylation for energy generation even in the presence of oxygen, namely aerobic glycolysis or the “Warburg effect”.^2^ Previous findings suggest that the high glucose consumption by cancer cells results in metabolic restriction of T cells and thereby contributing to immunosuppression and cancer progression.^3^ Whether the cell-extrinsic nutrient competition or the cell-intrinsic reprogramming leads to dysregulated pro-tumorigenic immune cell metabolism is still a matter of debate.

In an elegant approach, the authors used ^18^F-fluorodeoxyglucose (FDG) which is a common tracer in positron emission tomography (PET) imaging to measure glucose uptake and its differential role in cancer and cancer-related immune cells. In a first step, the study demonstrated that higher than normal glucose uptake is a hallmark of cancer-associated immune cells by comparing the FDG uptake of CD45 expressing immune cells in the tumor environment and splenocytes. Indeed, the glucose uptake was significantly higher in these cells than in the malignant cells characterized by missing CD45 expression. To further explore the nutrient partitioning within the subpopulations of cancer-related immune cells, Reinfeld et al. detected the FDG avidity of CD4^+^/CD8^+^ T-lymphocytes and other non-T CD45^+^ immune cells. Compared with the glucose uptake of cancer cells, the T-lymphocytes are characterized by a similar glucose uptake level, which is significantly higher than that of resting T-cells in the spleen. Consequently, T-cells in TME are not under glucose deprivation due to nutrient competition with cancer cells. What is more, the non-T CD45^+^ immune cells, more specifically, the CD11B^+^ myeloid cells, including the monocytic myeloid-derived suppressor cells (M-MDSCs) that are monocytes characterized by its immunosuppressive activity and tumor-promoting function and the tumor-associated macrophages (TAMs), are found to have the highest FDG uptake levels. Such findings are also in line with the results of the extracellular flux assays, where TAMs maintain the highest extracellular acidification rate (ECAR) and mitochondrial oxygen consumption rate (OCR). The essential role of glucose was also corroborated by gene enrichment analysis demonstrating high expression levels of genes involved in glucose metabolisms like glucose transporters and catalytic genes important for glucose phosphorylation. Taken together, M-MDSCs and TAMs rather than cancer cells metabolize most glucose per cell in TME.

To understand the cell-intrinsic mechanisms underlying the nutrient partitioning in TME, Reinfeld et al. focused on the mTORC1 signaling pathway, which has been reported to be closely associated with the metabolic reprogramming of cancer cells.^4^ By checking the level of phosphorylated ribosomal protein S6 (pS6) as a downstream of mTORC1 signaling, the authors observed that mTORC1 is highly activated in TAMs compared with other cell populations in TME. Moreover, after treating tumor-bearing mice with the specific mTORC1 inhibitor rapamycin, the glucose uptake of myeloid cells and cancer cells were significantly suppressed. In extracellular flux assays, mTORC1 inhibition by rapamycin significantly decreased the ECAR and OCR of myeloid cells but not cancer cells and T cells. Consistently, the expression of HK2, the key kinase catalyzing glucose phosphorylation in glycolysis, was also found to be downregulated in TAMs after rapamycin treatment. These findings further indicate that mTORC1 plays an important role in the glucose metabolism in TME.

When glucose is preferentially utilized by the tumor-related immune cells, the question arises: what is the energy source of cancer cells in this setting? The upregulation of genes related to glutamine metabolism like *Mycn* and *Atf4* among others suggested an important role of glutamine metabolism in the cancer cells. To prove the role of glutamine in the cancer cells, the authors studied glutamine uptake by measuring in vivo avidity for ^18^F-(2*S*,4*R*)4-fluoroglutamine (^18^F-Gln). In contrast to the immune cells which depend on glucose for their energy production, a greater ^18^F-Gln avidity in cancer cells was observed. Interestingly, when using a small-molecule inhibitor called V9302 to shut down the glutamine transport into the cells, increased glucose uptake levels were observed in both cancer cells and immune cells, which suggests that the role of amino acid as energy source can be substituted by glucose. In turn, this would result in glucose competition between malignant cells and immune cells which might modulate immunosuppression. In line with this argumentation, glutamine blockade has already been considered as a potential strategy for reversing immunosuppression.^5^

Even in cancer cell-derived glutamine metabolism, the mTOR pathway emerged as an important pathway of metabolic programming. Notably, rapamycin treatment markedly inhibits the uptake of ^18^F-Gln in cancer cells.

**Fig. 1:**
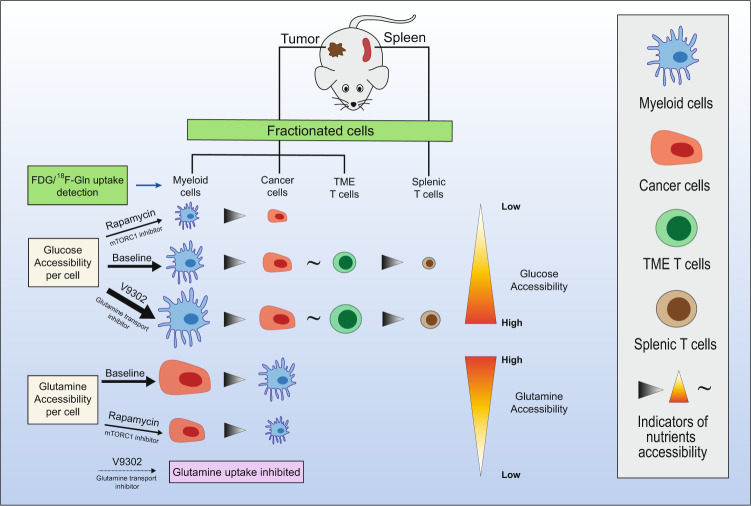
The mTORC1-driven metabolic reprogramming of distinct cell populations in TME. On the baseline level, the myeloid cells exhibit the greatest glucose uptake, followed by cancer cells and T cells in TME; while the cancer cells have the highest glutamine uptake level. All the cell populations in TME show higher nutrient uptake level than the normal splenic T cells. The inhibition of mTORC1 by rapamycin decreases the uptake of glucose and glutamine in all cell populations, and the blockade of glutamine transport induces upregulation of glucose uptake which might induce competition for glucose and reversal of immune suppression. The width of arrows, size of the cells, and the direction of the triangles represent the nutrient uptake accessibility (from high to low). The symbol “~” represents similar nutrient accessibility.

In conclusion, the results of this study indicate that the suppression of immune cells to eliminate cancer cells is not caused by cell-extrinsic nutrient competition. Instead, driven by mTORC1, the cell-intrinsic metabolic reprogramming that immune cells utilize glucose and cancer cells utilize amino acids as well as partly lipids to produce ATP plays an important role in generating a pro-tumorigenic immunosuppressive microenvironment (Fig. 1). This research not only highlights the feasibility of an elaborate radioisotope tracer method in the study of cancer metabolism, but also makes an important conceptual breakthrough that challenges the presumption that cancer cells induce nutrient deficiency and dysfunction of immune cells in TME by competing with them for glucose. Based on the dependence of cancer cells on glutamine, to improve PET imaging strategy might be able to provide more precise information for clinical cancer management. Furthermore, interfering with glutamine metabolism in cancer cells and thus making cancer cells more dependent on glucose for energy production might also be an interesting therapeutic avenue in the future.
